# Nutrition and Growth of Preterm Neonates during Hospitalization: Impact on Childhood Outcomes

**DOI:** 10.3390/nu16020218

**Published:** 2024-01-10

**Authors:** Antonios K. Gounaris, Rozeta Sokou

**Affiliations:** 1 Neonatal Intensive Care Unit, University Hospital of Larissa, 413 34 Larissa, Greece; angounaris@med.uth.gr; 2 Neonatal Intensive Care Unit, Nikea General Hospital “Agios Panteleimon”, 184 54 Piraeus, Greece

## 1. Introduction

The Special Issue has been completed with the publication of 13 review and research articles. Throughout the content of the articles and their relevant literature, two major themes emerged in our opinion.

Firstly, it is evident that the change in feeding protocols after the year 2000, especially for very premature neonates (VPNs), and despite some ongoing discussions that these changes generated, contributed significantly to improvements in survival and long-term morbidity even of neonates on the “grey zone” of viability (22–24^+6^ weeks) [1,2,3]. This is a monumental positive change in the treatment of premature neonates post 2000 that constitutes in our opinion a separate third era in Neonatology, rather than being a part of the second era. The adoption of this definition by Perinatal Societies would help to focus perinatal care on the growth and development of VPNs during hospitalization.

The second issue emerging in our opinion is that the time has come for the implementation of universal neonatal feeding protocols, based on the 2021 and 2022 ESPGHAN guidelines [4,5]. Despite some valid criticisms from eminent colleagues regarding the paucity of large-scale studies confirming the long-term outcomes of those policies, in our opinion, the wealth of studies showcasing the integral role of improved nutrition for long-term VPN prognosis should not be ignored by the scientific community. The choice to wait for large-scale studies is not a neutral one, as it will further entrench the huge inequalities in neonatal nutritional care currently observed between countries or even within neonatal units of the same country, with the associated disparity of outcomes.

Two studies explored growth patterns with the monitoring of head circumference (HC) and weight trajectory of VPNs. Lin et al. (contribution 1) showed an association between feeding progress in the first 56 days of life and HC at term and corrected ages of 6, 12 and 24 months, as well as neurodevelopment at 24 months corrected age. Infants showing slow feeding progress had a high risk of stunted HC growth or even microcephaly with associated neurodevelopmental disorders in early childhood. In the study of Peter et al. (contribution 2), there was an evaluation of a group of very-low-birth-weight (VLBW) infants that required surgical intervention and colostomy formation because of either necrotizing enterocolitis (NEC), spontaneous intestinal perforation (SIP) or meconium-related ileus (MI). It was found that the duration of inflammation and their growth velocity during hospitalization, as evidenced by their HC and weight, had a significant impact on the risk of cholestasis and adverse neurodevelopmental outcomes at 24 months irrespective of the etiology of the enteral surgery. These two studies further confirm the prevailing consensus that the delay of appropriate nutrition and growth in the neonatal period and up to 40 weeks corrected gestational age is detrimental to neurodevelopment at 24 months of age [6,7,8,9]. In the cases requiring surgical intervention, aiming to reduce ongoing inflammation, the early reintroduction of feeding and nutritional supplementation to aid in catch-up growth will help minimize those observed adverse effects. 

Along the same vein, in a review article, Kosmeri et al. (contribution 3) collate and present the current knowledge around the nutritional needs of fetal growth-restricted (FGR)/small for gestational age (SGA) premature neonates of singleton and multiple pregnancies. They note from the reviewed literature that premature FGR/SGA babies experience cumulative nutritional deficiencies because of a multitude of factors: malnutrition at the fetal stage, comorbidities in the first few weeks of life, delayed initiation and slow advancement of enteral nutrition [10,11]. This finding is more common in neonates born < 29 weeks and is confirmed by the latest study of Sériès et al. [6].

From those findings, the writers conclude that FGR/SGA VPNs require more aggressive feeding in the neonatal period in order to make up for these nutritional deficits. 

Two studies focus on the methods of monitoring and evaluating VPN growth during their NICU stay. Kakatsaki et al. (contribution 4) compared the prevalence of SGA and extrauterine growth restriction (EUGR) among extremely and very preterm neonates (GA < 32 weeks) using the Fenton13 and Intergrowth-21 growth charts. They showed a significant difference in the prevalence of neonates meeting the SGA and EUGR definitions using these two different charts, something that has been confirmed by separate studies [12,13,14,15,16] and that, in practice, hinders the implementation of more universal clinical and feeding practices for VPNs. It is necessary, according to the authors, to determine a growth chart that enables the appropriate optimal monitoring of these neonates.

In an opinion piece, Gounaris et al. (contribution 5) argue that the variable definitions of the terms intrauterine growth restriction (IUGR), extrauterine growth restriction (EGR) and postnatal growth failure (PGF) in the international literature directly contribute to the absence of a universally accepted feeding and growth strategy for this infant population. In their opinion, the term EGR is more appropriate compared to PGF, as it focuses on a defined period of growth, up to 40 weeks corrected gestational, age that usually corresponds with the duration of hospital stay and during which nutritional interventions have maximum impact. In contrast, PGF refers to growth during an ill-defined period that can be extended up to the first year of life. Considering which growth paradigm should be considered safe, they argue that based on studies [17] showing that VPN growth > 10th centile at 36 weeks corrected gestational age (CGA) or at discharge did not increase neurodevelopmental risks, and that aiming for growth above the traditional definition of growth restriction (<10th centile for CGA) is both a safe and feasible target until a more precise one is determined through large scale studies. In terms of the adoption of a universal feeding policy, in the authors opinion the latest ESPGHAN guidelines from 2021 and 2022 can be used as a blueprint and a stepping stone to that direction [4,5].

Over the last few years, multiple studies have highlighted the benefits of breast milk for premature neonates, something reflected in the five studies focusing on breast feeding and breast milk. Seliga-Siwecka et al. (contribution 6) did not find a significant difference between targeted and standard breast milk fortification in terms of growth velocity during NICU stay, as reflected in HC, length and weight gain. This goes on to show that even if targeted breast milk fortification is not technically feasible, standard fortification has similar effects on VPN growth.

In the study by Gialeli et al. (contribution 7), it was found that donor breast milk (DBM) from mothers of premature neonates resulted in a significantly improved intake of protein (*p* = 0.006) and improved weight gain (*p* = 0.019) compared to term DBM, a finding that strengthens the argument that term DBM should be fortified in a targeted way when administered to VPNs. The study of Sokou et al. (contribution 8) confirmed the low levels of breast feeding among neonates hospitalized in NICUs, which are far lower than the targets set out by the WHO and CDC [18,19], and defined the causative factors behind them.

In a systematic review by Dimitroglou et al. (contribution 9), it was noted that SARS-CoV-2 infection promotes an IgA immune response in breast milk, whereas COVID vaccinations mainly caused an IgG response. In both cases, the levels of IgG immunoglobulins in maternal blood are closely correlated with immunoglobulin levels in breast milk. The authors conclude that breast milk from mothers who have been infected or received a vaccination against the virus could help protect their babies during the pandemic.

A review by Sokou et al. (contribution 10) explored the ability of women affected by long-term conditions to breast feed. Cumulative evidence shows that women with long term health conditions achieve lower levels of breast feeding compared to healthy controls as a result of a multitude of contributing factors. This study examines in detail how each of the main long-term health morbidities affects exclusive breast-feeding levels. Interventions that support both maternal health and caregiving abilities will help reverse this trend.

Two reviews focus on the short- and long-term effects of BPD, one of the major morbidities of prematurity. In a literature review by Karatza et al. (contribution 11), the authors conclude that this population should receive sufficient calories and nutrients, both during and after their NICU stay, in order to achieve an adequate level of growth that will support lung alveolarization. The precise monitoring of growth both during and after discharge from hospital is necessary according to the authors as minimizing growth restriction will help improve lung function.

Likewise in their review, Briana and Malamitsi (contribution 12) examined the relationship between the parameters of hospital stay of extremely/very premature neonates and their long-term respiratory prognosis. Long-term observational studies have concluded that extremely/very premature birth is significantly associated with Chronic Obstructive Pulmonary Disease (COPD), something that respiratory physicians should be aware of. The authors here infer that the recent data underscore the significance of beneficial nutritional interventions in the neonatal period, for the long-term development of the lungs and their capacity to recover from damage [20,21]. The above findings could positively contribute to the long-term prognosis of extremely/very premature neonates. 

The effects of prematurity on later life were explored in the study of Wood et al. (contribution 13) by examining the correlation between gestational age (GA) and the metabolic and muscle function during adolescence using 3IP-MRS. It was found that GA at birth was predictive of oxidative skeletal muscle function (T1/2 PCr) in adolescence. This finding demonstrates, according to the authors, the persistence of the metabolic impact of prematurity on later life.

In conclusion, these 13 studies along with their literature provide enough support to the argument for the more ‘intense’ nutrition of VPNs in the neonatal period, including breast milk fortification and closely following ESPHGHAN guidelines. Especially in the cases of neonates that are IUGR, SGA, born <29 weeks GA or affected by major morbidities such as BPD, strict adherence to guidelines with nutritional deficit replacement is necessary as it can make a positive difference in the lives of these neonates in years to come. 
